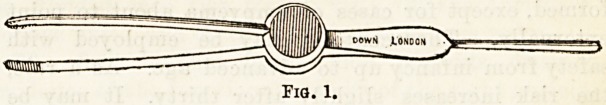# Diseases of the Ear

**Published:** 1894-09-29

**Authors:** P. Macleod Yearsley

**Affiliations:** Aural Surgeon and Surgeon to Farringdon General Dispensary, Assistant Demonstrator of Anatomy, Curator of Museum, and Demonstrator of Surgical Pathology to Westminster Hospital


### Medical Progress and Hospital Clinics.

[*The Editor will be glad to receive offers of co-operation and contributions from members of the profession. All letters should be addressed to* The Editor, The Lodge, Porchester Square, London, W.]

### DISEASES OF THE EAR.

By P. Macleod Yearsley, F.R.C.S.Eng., Aural Surgeon and Surgeon to Farringdon General Dispensary, Assistant Demonstrator of Anatomy, Curator of Museum, and Demonstrator of Surgical Pathology to Westminster Hospital.

### V.—The Artificial Membrana Tympani.

There has always been some difference of opinion as to the value of the artificial membrana tympani, and it has received praise and blame in fairly equal quantities. Its injudicious use may certainly do harm, but when properly employed in suitable cases it will as certainly do good. I have little doubt that there are many persons suffering with perforation whom its application would greatly benefit, but, unfortunately, the great majority of these cases do not come under the notice of the aurist, and if they go to a doctor at all, his otological knowledge is either too deficient to allow of his recognising them, or his time is too much occupied to be spent in the perseverance necessary for success.

I propose in this article to mention some of the chief varieties of the appliance, and to describe in detail the use of one such, which I consider to possess the greatest advantage over other forms, and of which I have the most experience.

In his "Manual of Diseases of the Ear" Mr. Field gives a list of nineteen different artificial membranes which have been introduced from time to time, and, taking only those of most importance, this number may be reduced to five, viz.: (1) Yearsley's cotton-pellet; (2) Toynbee's solid indiarubber disc, fixed to a silver wire stem; (3) Field's compound disc of india-rubber, cotton-wool, and flannel; (4) Politzer's india-rubber tube as long as the meatus; and (5) Ward Cousins' hat-shaped membrane of boracic lint. Of these the earliest form is the simple cotton-wool pellet introduced by my late relative, Mr. James Yearsley, whose first account thereof may be found in the *Lancet* for July 1st, 1848; and although its modifications, particularly those of Ward Cousins and Field, have been of great use in the hands of their inventors, I think the original form must still hold its own as the simplest, and therefore the best. I have repeatedly employed it during the past six years, and as it is easily made and is inexpensive it is the one most likely to commend itself to the general practitioner.

The way in which an artificial membrane acts has been productive of some difference of opinion. Yearsley believed that the natural membrane, besides receiving vibrations, had an important action in supporting the ossicular chain, andthat the cotton-wool pellet restored this support when lost by perforation. Toynbee, on the other hand, thought that the drum-head confined the sonorous undulations to the tympanic cavity in order that they might be concentrated on the membrana fenestræ rotundæ, and that the artificial membrane restored this function. Probably there is right on both sides, but space is too limited to allow of its discussion. The former argument is strengthened by the fact that the appliance will act best when pressing at one particular spot of the injured membrane, a spot which can generally be found with a probe before introducing the artificial tympanum, and at which the support to the ossicular chain is apparently lost.

According to some authorities, the cases best suited for the treatment are those in which the drumhead is partially or wholly lost; but it will be found that in all varieties of perforation, from small holes to almost complete loss of the membrane, there will be cases in which the application of the artificial drum is attended with immediate benefit, while in others, apparently similar, no good can be obtained even after the most careful and persevering attempts. In fact, it is not possible to tell beforehand whether a case will be improved or otherwise. The presence of profuse otorrhœa is, of course, a bar to the treatment; but a moderate and sweet discharge is no prohibition, and the application of a medicated drum will often be found to have a good effect upon it.

The advantages which may be claimed for the form introduced by Yearsley are four: (1) It is simple and effective, (2) the patient can make it for himself, (3) it can be made of any antiseptic and absorbent wool, and (4) its proper adjustment once found, it is easy of manipulation. It can be manufactured in a moment by taking a small wisp of wool, tying a cotton thread around its centre, and rolling it into a spherical shape. In order to apply it, it must first be moistened with warm glycerine, to which may be added a little carbolic acid or any other drug which the surgeon wishes to apply to the membrane. It is then introduced into the meatus with a small pair of forceps (Fig. 1), and

Fig. 1.

pushed down to the membrane. It is then gently moved with a probe into different positions until the precise spot at which it gives the necessary support is found, the hearing being frequently tested with the watch. It is this part of the manipulation which requires patience, but the benefit which may result is worth the expenditure of a little time, and when once the proper adjustment of the pellet is discovered, the patient can be easily taught to manipulate it for himself.

Success attained, there are certain simple precautions to be observed in the after use of the artificial membrana tympani, and these apply to all its varieties. (1) It should be at first only worn for an hour or so, until the patient is used to and tolerates it; (2) it should not be worn at night, especially if there be any discharge; and (3) should it give rise to any irritation, it must be discarded until such irritation has been overcome by treatment.

In conclusion, I would urge the general practitioner to make occasional trial of this treatment. With a little practice it is easy, and requires patience merely, and by its means he may be able to render less irksome the lot of many persons suffering from perforation and deafness.

## Figures and Tables

**Fig. 1. f1:**